# Decomposing and simplifying the Fracture Risk Assessment Tool—a module from the Taiwan-specific calculator

**DOI:** 10.1093/jbmrpl/ziae039

**Published:** 2024-03-23

**Authors:** Chia-Chun Li, I-Ting Liu, Tien-Tsai Cheng, Fu-Wen Liang, Zih-Jie Sun, Yin-Fan Chang, Chin-Sung Chang, Yi-Ching Yang, Tsung-Hsueh Lu, Li-Chieh Kuo, Chih-Hsing Wu

**Affiliations:** Institute of Allied Health Sciences, College of Medicine, National Cheng Kung University, 701 Tainan, Taiwan; Department of Family Medicine, College of Medicine, National Cheng Kung University, 701 Tainan, Taiwan; Department of Family Medicine, E-DA Hospital, 824 Kaohsiung, Taiwan; Department of Geriatric Medicine, E-DA Hospital, 824 Kaohsiung, Taiwan; School of Medicine, College of Medicine, I-Shou University, 840 Kaohsiung, Taiwan; Division of Rheumatology, Allergy and Immunology, Kaohsiung Chang Gung Memorial Hospital, 833 Kaohsiung, Taiwan; Department of Public Health, College of Health Sciences, Kaohsiung Medical University, 807 Kaohsiung, Taiwan; Division of Family Medicine, National Cheng Kung University Hospital Dou Liu Branch, 640 Yunlin, Taiwan; Department of Family Medicine, National Cheng Kung University Hospital, College of Medicine, National Cheng Kung University, 704 Tainan, Taiwan; Department of Family Medicine, National Cheng Kung University Hospital, College of Medicine, National Cheng Kung University, 704 Tainan, Taiwan; Department of Family Medicine, National Cheng Kung University Hospital, College of Medicine, National Cheng Kung University, 704 Tainan, Taiwan; Department of Family Medicine, College of Medicine, National Cheng Kung University, 701 Tainan, Taiwan; Department of Family Medicine, National Cheng Kung University Hospital, College of Medicine, National Cheng Kung University, 704 Tainan, Taiwan; Department of Public Health, College of Medicine, National Cheng Kung University, 701 Tainan, Taiwan; Institute of Allied Health Sciences, College of Medicine, National Cheng Kung University, 701 Tainan, Taiwan; Department of Occupational Therapy, College of Medicine, National Cheng Kung University, 701 Tainan, Taiwan; Department of Family Medicine, College of Medicine, National Cheng Kung University, 701 Tainan, Taiwan; Department of Family Medicine, National Cheng Kung University Hospital, College of Medicine, National Cheng Kung University, 704 Tainan, Taiwan; Institute of Gerontology, College of Medicine, National Cheng Kung University, 701 Tainan, Taiwan

**Keywords:** FRAX®, fracture risk assessment tool, risk of fracture

## Abstract

The Fracture Risk Assessment Tool (FRAX®) is a widely utilized country-specific calculator for identifying individuals with high fracture risk; its score is calculated from 12 variables, but its formulation is not publicly disclosed. We aimed to decompose and simplify the FRAX® by utilizing a nationwide community survey database as a reference module for creating a local assessment tool for osteoporotic fracture community screening in any country. Participants (*n* = 16384; predominantly women (75%); mean age = 64.8 years) were enrolled from the Taiwan OsteoPorosis Survey, a nationwide cross-sectional community survey collected from 2008 to 2011. We identified 11 clinical risk factors from the health questionnaires. BMD was assessed via dual-energy X-ray absorptiometry in a mobile DXA vehicle, and 10-year fracture risk scores, including major osteoporotic fracture (MOF) and hip fracture (HF) risk scores, were calculated using the FRAX®. The mean femoral neck BMD was 0.7 ± 0.1 g/cm^2^, the T-score was −1.9 ± 1.2, the MOF was 8.9 ± 7.1%, and the HF was 3.2 ± 4.7%. Following FRAX® decomposition with multiple linear regression, the adjusted *R*^2^ values were 0.9206 for MOF and 0.9376 for HF when BMD was included and 0.9538 for MOF and 0.9554 for HF when BMD was excluded. The FRAX® demonstrated better prediction for women and younger individuals than for men and elderly individuals after sex and age stratification analysis. Excluding femoral neck BMD, age, sex, and previous fractures emerged as 3 primary clinical risk factors for simplified FRAX® according to the decision tree analysis in this study population. The adjusted *R*^2^ values for the simplified country-specific FRAX® incorporating 3 premier clinical risk factors were 0.8210 for MOF and 0.8528 for HF. After decomposition, the newly simplified module provides a straightforward formulation for estimating 10-year fracture risk, even without femoral neck BMD, making it suitable for community or clinical osteoporotic fracture risk screening.

## Graphical Abstract

**Graphical Abstract:**
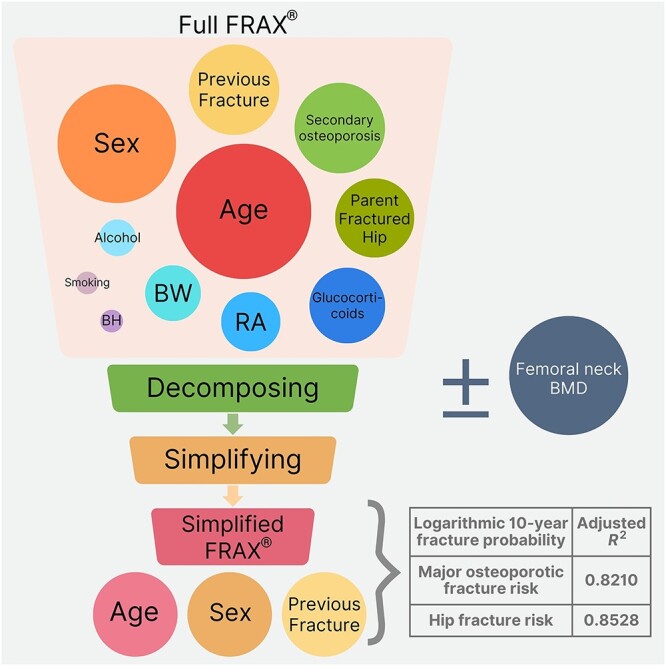

## Introduction

Osteoporosis is a common age-related disease that causes bone fragility and osteoporotic fractures due to low bone mass and microarchitectural deterioration of bone tissue.^(^^1^^)^ According to a World Health Organization (WHO) report, osteoporotic fracture is the primary cause of disability-adjusted life years, followed by hypertension.^(^^2^^)^ According to the literature review, anti-osteoporosis medicines could significantly lower mortality, particularly among older adults, hip fracture (HF) patients, and those who take bisphosphonate.^(^^3–5^^)^ Osteoporosis is a silent disease because it usually presents without symptoms until fracture occurs. Prevention of the first fracture is challenging, but we can prevent the second and other future fractures. Preventing either primary or secondary osteoporotic fractures is the best way to decrease the extensive burden of osteoporosis.

The widely accepted gold standard for measuring BMD and identifying osteoporosis is DXA.^(^^6^^)^ Several clinical risk factors can increase the sensitivity of a predictive model for hip and other osteoporotic fractures based solely on BMD.^(^^7^^)^ The Fracture Risk Assessment Tool (FRAX®) is a commonly used method for estimating the probability of HF or major osteoporotic fracture (MOF) within the next decade.^(^^8^^)^ Country-specific calculator-associated intervention thresholds have been developed for countries such as the United Kingdom, Japan, Switzerland, Taiwan, and the United States.^(^^9–14^^)^

The FRAX® still reveals up to 15-fold differences in HF incidence between very high- and low-incidence countries.^(^^15^^)^ Despite the FRAX® being available for the Chinese population, in the Kanis et al. study, the 10-year HF probability in Taiwan was greater than that in Hong Kong and China.^(^^15^^)^ Cheung et al. showed that the incidence of HF in women and men in Taiwan is greater than that in other Asian countries.^(^^16^^)^ To our knowledge, research using the FRAX® to estimate the probability of osteoporotic fracture in Asia is inadequate. A history of falling and recurrent falls are essential predictors of major osteoporotic fractures, even in Taiwan.^(^^17^^)^ Furthermore, Vandenput et al.’s systematic review revealed new risk factors for fracture, such as diabetes history and fall history.^(^^18^^)^ In contrast, the FRAX® does not account for falls or other factors that affect fracture risk, such as bone turnover markers and vertebral BMD. Therefore, the country-specific 10-year probability of osteoporotic fracture needs to be re-estimated and validated using the local population for a better prediction model. Osteoporosis Research Ltd developed the FRAXplus®, which allows users to add probable risk factors to improve the FRAX®, such as osteoporotic fracture and fall history and information on lumbar spine and trabecular bone scores.^(^^19^^)^ Although adding clinical risk factors can improve FRAX® accuracy, too many factors will not only increase the difficulty of implementation but also reduce the feasibility of primary screening.

The primary objective of this real-world study was to decompose the FRAX® algorithm and identify significant clinical risk factors via the nationwide community survey database for the Taiwanese population as a module. The secondary objective was to simplify the FRAX® for community or clinical screening more straightforwardly.

## Materials and methods

### Study population

The Taiwan OsteoPorosis Survey (TOPS) is a database formed by a cross-sectional community survey conducted by the Taiwanese Osteoporosis Association (TOA) between 2008 and 2011.^(^^20^^,^^21^^)^ This database, covering 450 surveys at 104 sites around Taiwan, was generated by using a mobile DXA vehicle (Explorer; Hologic, Inc, Waltham, MA) operated by the International Society for Clinical Densitometry (ISCD)-certified radiology technician.^(^^22^^)^ The study sites, which included both urban and rural areas, were chosen at random and were dispersed equally. All participants who were voluntarily advised to take part in the survey; however, those who could not use the DXA or had both hips previously broken or replaced were not included. The survey was approved by the Institutional Review Board of Chang Gung Memorial Hospital (102-1878B) and National Cheng Kung University Hospital (B-ER-108-148).

### Data collection and measurements

A well-trained nurse conducted interviews with each participant to complete the health questionnaires, which included 11 clinical risk factors for the FRAX®.^(^^23^^)^ The BMD (optional) was measured via DXA, including the lumbar spine and hip regions, and converted into T-scores. It is not advisable to employ measurements other than femoral neck BMD or T-scores derived via DXA for utilization in the FRAX® standard.^(^^24^^)^ The National Health and Nutrition Examination Survey (NHANES III) served as the foundation for the T-score reference value.^(^^25^^)^

The FRAX® (https://frax.shef.ac.uk/FRAX/index.aspx) can generate 2 types of 10-year fracture risk probabilities, HF and MOF (including fractures of the hip, spine, forearm, and shoulder), either with or without BMD, based on femoral neck BMD and 11 clinical risk factors.

### Statistical analysis

This study aimed to decompose and simplify the 10-year fracture risk probability of HFs and MOFs with 12 FRAX® clinical risk factors. First, an extended scatterplot matrix was used to plot all continuous factors of the FRAX® clinical risk factors, including age, height, weight, BMD, and MOF/HF risk, revealing a general relationship with the 10-year fracture risk probability of MOF/HF patients. Second, the relative impact of each FRAX® clinical risk factor was evaluated through multiple linear regression analysis. Decision tree analysis was subsequently performed using the 10-year probability as the dependent variable and 12 FRAX® clinical risk factors as the independent predictors to explore the importance of the FRAX® score with and without BMD. We used 2 studies that determined the Taiwan intervention threshold to classify the 10-year fracture risk probability of HFs and MOFs.^(^^13^^,^^26^^)^

Statistical analyses were carried out using *t* test to compare continuous variables and chi-square tests to compare categorical variables. To indicate statistical significance, a two-sided probability of 0.05 was used. All the data were analyzed with SAS® software version 9.4 (SAS Institute, Inc, Cary, NC, United States).

## Results

A total of 18 559 participants participated in the survey between 2008 and 2011. Due to the FRAX® being applicable only to individuals aged between 40 and 90 years and without a history of medical treatment for osteoporosis, only 16 384 participants were enrolled in this study. As shown in Table 1, the participants were predominantly women (75.7%), with a mean age of 64.8 ± 10.6 years. The mean height was 157.0 ± 7.6 cm, the mean weight was 59.6 ± 10.2 kg, and the mean BMI was 24.1 ± 3.6 kg/m^2^; these patients were considered to have a normal weight according to WHO recommendations.^(^^27^^)^ The mean femoral neck BMD was 0.7 ± 0.1 g/cm^2,^ and the T-score was −1.8 ± 1.1, indicating osteopenia. Generally, the 10-year fracture risk in women was greater than that in men, regardless of the presence of MOF or HF. This table was shown in Supplementary Table S1. Most of the participants were at low risk according to the International Oncology Group (IOF)/TOA criteria, which was also consistent with Liu’s criteria, but women were at higher risk according to Liu’s criteria.^(^^26^^,^^28^^,^^29^^)^ The intervention thresholds included drug and nondrug treatment for osteoporosis to avoid nontraumatic fractures.

**Table 1:** Baseline characteristics of the study population.

	Total	Men	Women	*P* value
Case number (%)	16 384(100.0)	3983(24.3)	12 401(75.7)	
FRAX® clinical risk factors				
Age, years	64.8 ± 10.6	68.7 ± 10.2	63.6 ± 10.4	<.0001
40 ~ 49 (%)	1229(7.5)	167(4.2)	1062(8.6)	<.0001
50 ~ 64 (%)	6679(40.8)	1086(27.3)	5593(45.1)	
65 ~ 74 (%)	5148(31.4)	1502(37.7)	3646(29.4)	
Above 75 (%)	3328(20.3)	1228(30.8)	2100(16.9)	
Height, cm	157.0 ± 7.6	165.1 ± 6.2	154.4 ± 5.9	<.0001
Weight, kg	59.6 ± 10.2	66.1 ± 10.2	57.4 ± 9.3	<.0001
BMI, kg/m^2^	24.1 ± 3.6	24.2 ± 3.3	24.1 ± 3.7	0.0272
Femoral neck BMD, g/cm^2^	0.7 ± 0.1	0.8 ± 0.1	0.7 ± 0.1	<.0001
Parent fractured hip (%)	1001(6.1)	236(5.9)	765(6.2)	0.5764
Previous fracture (%)	518(3.2)	97(2.4)	421(3.4)	0.0026
Glucocorticoids use (%)	653(4.0)	113(2.8)	540(4.4)	<.0001
Rheumatoid arthritis (%)	672(4.1)	113(2.8)	559(4.5)	<.0001
Secondary osteoporosis (%)	2087(12.7)	157(3.9)	1930(15.6)	<.0001
Current smoking (%)	545(3.3)	424(10.7)	121(1.0)	<.0001
Alcohol 3 or more units/day	173(1.1)	122(3.1)	51(0.4)	<.0001
FRAX® MOF risk with BMD, %	8.2 ± 6.4	5.4 ± 3.3	9.1 ± 6.9	<.0001
FRAX® HF risk with BMD, %	2.8 ± 4.2	2.0 ± 2.6	3.1 ± 4.6	<.0001
Lowest T-score, SD^a^	−1.8 ± 1.1	−1.4 ± 1.0	−2.0 ± 1.1	<.0001
> = −1.0	3931(24.0)	1453(36.5)	2478(20.0)	<.0001
−1.0 to −2.5	7569(46.2)	1968(49.4)	5601(45.2)	
<= − 2.5	4884(29.8)	562(14.1)	4322(34.9)	

FRAX®, Fracture Risk Assessment Tool; HF, hip fracture; MOF, major osteoporotic fracture. The data are presented as the *n* (%) for categorical variables or mean ± SD for continuous variables, unless indicated otherwise. The *P* value was calculated for continuous variables via the *t* test or for categorical variables via the *χ*2 test.

a The lowest T-score indicates the minimum T-score among the lumbar spine, femoral neck, or total hip.

**Figure 1:**
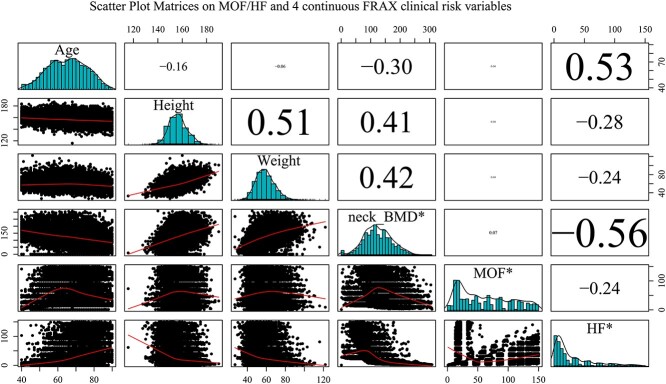
Scatter plot matrices on MOF/HF and 4 continuous FRAX clinical risk variables. *neck_BMD, femoral neck BMD; MOF, major osteoporotic fracture; HF, hip fracture.

As shown in Figure 1, the histograms (frequency distributions) of all the variables were depicted on the main diagonal, while the range of each variable was depicted on the side axes of the plot. The corresponding linear correlations of each plot were shown above the diagonal, and the font size was scaled based on the value, with larger font sizes signifying higher correlation values. The correlations between MOF probability and 4 continuous FRAX® clinical risk factors revealed a nonlinear correlation, which was converted by log-transformation of the 10-year MOF and HF probabilities in a regression model.

To decompose the fracture risk prediction model, a linear regression analysis was performed on 12 FRAX® clinical risk factors to estimate the FRAX® score. Table 2 shows the results of the multiple linear regression analysis for the logarithmic association between the 10-year FRAX® MOF and HF risk. After controlling for the associated factors, the adjusted *R*^2^ values for MOF/HF were 0.9206/0.9376 for FRAX® with BMD and 0.9538/0.9554 for the FRAX® without BMD. With respect to the 10-year FRAX® fracture risk in the BMD model, femoral neck BMD and height were negatively related, but only the difference in weight was statistically negatively significant in the FRAX® without BMD.

**Table 2:** Multiple linear regression analysis of the decomposition of FRAX® based on the logarithmic 10-year MOF and HF probabilities.

	With BMD (*n* = 16 127)		Without BMD (*n* = 16 384)	
	MOF risk	HF risk	MOF risk	HF risk
	β (95% CI)	β (95% CI)	β (95% CI)	β (95% CI)
Multiple *R*^2^ value	0.9207	0.9377	0.9538	0.9555
Adjusted *R*^2^ value	0.9206	0.9376	0.9538	0.9554
Femoral neck BMD	−2.84(−2.86 to −2.81)	−7.64(−7.69 to −7.59)	-	-
Age	0.03(0.03-0.03)	0.06(0.06-0.06)	0.06(0.06-0.06)	0.11(0.11-0.11)
Sex	0.31(0.30-0.32)	−0.24(−0.25 to −0.22)	0.57(0.56-0.58)	0.56(0.55-0.57)
Height	−0.01(−0.01 to −0.01)	−0.003(−0.004 to −0.002)	0.01(0.01-0.01)	0.02(0.02-0.02)
Weight	0.01(0.01-0.01)	0.004(0.003-0.005)	−0.01(−0.01 to −0.01)	−0.03(−0.03 to −0.03)
Parent fractured hip	0.64(0.63-0.65)	0.35(0.33-0.37)	0.62(0.61-0.63)	0.47(0.46-0.49)
Previous fracture	0.45(0.44-0.47)	0.41(0.38-0.44)	0.59(0.58-0.61)	0.75(0.72-0.77)
Glucocorticoids use	0.41(0.39-0.43)	0.54(0.51-0.57)	0.45(0.43-0.46)	0.66(0.64-0.69)
Rheumatoid arthritis	0.27(0.25-0.29)	0.35(0.32-0.38)	0.27(0.25-0.28)	0.42(0.39-0.44)
Secondary osteoporosis	0.01(−0.002 to 0.02)	0.01(−0.001 to 0.03)	0.33(0.32-0.33)	0.52(0.51-0.53)
Current smoking	0.08(0.06-0.10)	0.41(0.38-0.44)	0.12(0.10-0.13)	0.33(0.30-0.35)
Alcohol consumption	0.28(0.25-0.31)	0.40(0.35-0.45)	0.27(0.25-0.30)	0.45(0.41-0.49)

FRAX®, Fracture Risk Assessment Tool; HF, hip fracture; MOF, major osteoporotic fracture.

We used a stratified analysis to examine the impact of sex and age. After controlling for 12 FRAX® clinical risk indicators, the 10-year MOF and HF probabilities in the model with BMD were more predictive of women than men (the adjusted *R*^2^ values were 0.9407 and 0.9529 for women and 0.8746 and 0.9065 for men); however, the 10-year MOF probabilities in the model without BMD were similar according to sex (Supplementary Tables S2 and S3). Femoral neck BMD and height were negatively related to MOF and HF risk in the model with BMD, but weight was not correlated with BMD. According to the stratified analysis by age, the 10-year MOF probability was more predictive of progression in younger adults, either with or without respect to BMD (Supplementary Tables S4 and S5). The 10-year MOF and HF probability in the model with BMD were more predictive of younger patients (the adjusted *R*^2^ values were 0.9529 and 0.9805), but the 10-year fracture probability in the model without BMD was similar. For MOF, the risk in the model with BMD still showed a negative relationship with femoral neck BMD and height, and weight was the only negative factor in the model without BMD. However, for HF risk, femoral neck BMD, sex, height, and weight were negatively related, and similar results were found for the FRAX® without BMD.

As shown in Table 3, the decision tree highlighted the importance of 12 FRAX® risk factors, with femoral neck BMD being the most important factor in predicting 10-year fracture risk in the model with BMD, followed by age. In contrast, age was the most important factor for predicting 10-year fracture risk in the model without BMD, followed by sex in the MOF group and weight in the HF group.

**Table 3:** Differential importance levels of 12 FRAX® clinical risk factors according to decision tree analysis (*n* = 16 384).

MOF risk		HF risk	
With BMD	Without BMD	With BMD	Without BMD
Femoral neck BMD (1.00)	Age (1.00)	Femoral neck BMD (1.00)	Age (1.00)
Age (0.61)	Sex (0.80)	Age (0.75)	Weight (0.34)
Sex (0.43)	Secondary osteoporosis (0.41)	Glucocorticoids (0.18)	Secondary osteoporosis (0.22)
Parent fractured hip (0.34)	Previous fracture (0.41)	Parent fractured hip (0.15)	Sex (0.16)
Previous fracture (0.25)	Parent fractured hip (0.33)	Previous fracture (0.12)	Glucocorticoids (0.15)
Glucocorticoids (0.16)	Glucocorticoids (0.29)	Rheumatoid arthritis (0.06)	Previous fracture (0.14)
Weight (0.10)	Rheumatoid arthritis (0.19)	Sex (0.06)	Rheumatoid arthritis (0.13)
Rheumatoid arthritis (0.08)	Weight (0.14)	Current smoking (0.05)	Parent fractured hip (0.05)
	Alcohol consumption (0.05)		Current smoking (0.05)

FRAX®, Fracture Risk Assessment Tool; HF, hip fracture; MOF, major osteoporotic fracture.

As shown in Table 4, we applied 3 important clinical risk factors, namely, age, sex, and previous fracture, to simplify the FRAX®, and the adjusted *R*^2^ value was greater than 0.8 for all participants (0.8210 for MOF and 0.8528 for HF) and for women (0.8375 for MOF and 0.8565 for HF). The definition of “previous fracture” was the same as that for the FRAX®. When the 4 important clinical risk factors (age, sex, parent fractured hip, and previous fracture) were applied (Supplementary Table S6), the adjusted *R*^2^ values were 0.8771 for MOF and 0.8655 for HF in the total population. When femoral neck BMD was added to the models, the adjusted *R*^2^ values increased substantially (Supplementary Tables S7 and S8).

**Table 4:** Simplified FRAX® without BMD was used to evaluate 3 primary clinical risk factors with a logarithmic 10-year fracture probability.

	Total participants (*n* = 16 384)		Men (*n* = 3983)		Women (*n* = 12 401)		<65 years old (*n* = 7908)		> = 65 years old (*n* = 8476)	
	MOF risk	HF risk	MOF risk	HF risk	MOF risk	HF risk	MOF risk	HF risk	MOF risk	HF risk
	β (95% CI)	β (95% CI)	β (95% CI)	β (95% CI)	β (95% CI)	β (95% CI)	β (95% CI)	β (95% CI)	β (95% CI)	β (95% CI)
Multiple *R*^2^ value	0.8210	0.8528	0.7631	0.8438	0.8375	0.8565	0.7193	0.7342	0.7187	0.6168
Adjusted *R*^2^ value	0.8210	0.8528	0.7630	0.8437	0.8375	0.8565	0.7192	0.7341	0.7186	0.6167
Intercept	−1.61 (−1.63 to −1.58)	−6.41 (−6.46 to −6.37)	−1.28 (−1.34 to −1.23)	−6.47 (−6.57 to −6.38)	−1.87 (−1.90 to −1.84)	−6.56 (−6.61 to −6.50)	−2.45 (−2.51 to −2.39)	−7.45 (−7.55 to −7.35)	−0.33 (−0.40 to −0.26)	−4.01 (−4.13 to −3.90)
Age	0.06 (0.06-0.06)	0.11 (0.11-0.11)	0.04 (0.04-0.04)	0.10 (0.10-0.10)	0.06 (0.06-0.06)	0.11 (0.11-0.11)	0.07 (0.07-0.07)	0.13 (0.13-0.13)	0.04 (0.04-0.04)	0.08 (0.08-0.08)
Sex	−0.60 (−0.61 to −0.58)	−0.59 (−0.61 to −0.57)	-	-	-	-	−0.44 (−0.46 to −0.42)	−0.58 (−0.61 to −0.55)	−0.68 (−0.69 to −0.67)	−0.59 (−0.61 to −0.57)
Previous fracture	0.73 (0.70-0.75)	0.91 (0.87-0.95)	0.72 (0.67-0.77)	0.88 (0.79-0.97)	0.71 (0.68-0.74)	0.91 (0.86-0.96)	0.87 (0.83-0.92)	1.26 (1.19-1.34)	0.66 (0.63-0.68)	0.76 (0.72-0.81)

FRAX®, Fracture Risk Assessment Tool; HF, hip fracture; MOF, major osteoporotic fracture.

Multicollinearity was detected by using a correlation matrix and variance inflation factor (VIF). As shown in Supplementary Table S9, the correlation coefficients of femoral neck BMD and age with MOF risk according to the model were between 0.6 and 0.8, with a strong correlation, and between height and weight were between 0.2 and 0.4. Only age was positively correlated with the FRAX® score. The VIFs ranged from 1.1 ~ 1.5, indicating a moderate correlation.

We also attested to this simplified module in a compatible cohort for external validation. This cohort consisted of individuals aged 40 years and older from Yunlin County and Tianliao District in Taiwan during the period from 2009 to 2010.^(^^26^^)^ The characteristics of the two cohorts were shown in the Supplementary Table S10. The model interpretability (*R*^2^) of the compatible cohort was also greater than 60% for better goodness of fit, and the root-mean-square error was 0.47 (less than 0.5) despite differences in age distributions and sex compositions between cohorts. These preliminary findings will undergo further refinement through a series of external validation studies in the near future.

## Discussion

The FRAX®, a fracture risk prediction tool, has been validated in various scenarios. This study conducted a linear regression analysis to elucidate the regression coefficients of clinical risk factors within the FRAX® that effectively broke down its components. Furthermore, a decision tree analysis was employed to simplify the original 12 FRAX® factors. This study reconfirms the significance of 4 primary variables, femoral neck BMD, age, sex, and previous fracture history, as they exhibit high importance in fracture prediction. These findings are compatible with existing studies validating the FRAX®. Undoubtedly, age is a substantial factor in predicting subsequent fracture risk, which is consistent with the observations of Sambrook et al.^(^^30^^)^ The predictive power of previous fractures also aligns with the results reported by Kanis et al. and Kung et al.^(^^31^^,^^32^^)^

This study highlights femoral neck BMD as a robust predictor of fractures, although accessibility to DXA measurements may present challenges. In cases where the BMD is unavailable, incorporating other clinical risk factors could enhance the performance of the FRAX®. Interestingly, the FRAX® without BMD sometimes outperforms the FRAX® with BMD in identifying individuals who may benefit from a BMD measurement. However, it is essential to acknowledge that the clinical risk variables used in the FRAX® were initially derived from specific cohorts, which may not account for all ethnic disparities. Furthermore, the presence of recall bias in clinical risk factors must be acknowledged.^(^^33^^,^^34^^)^ Therefore, a simplified module that relies on relatively unbiased variables such as sex, age, and previous fracture history may offer more reliable results.

The FRAX®, introduced in 2008, was designed to assess fracture probability for individuals aged 40–90 years in primary care, considering clinical risk variables with or without BMD measurements.^(^^9^^)^ Numerous studies have leveraged FRAX® values for various purposes, including establishing treatment thresholds and assessing accuracy, among others. These studies have also constructed country-specific evaluation benchmarks.^(^^7^^,^^18^^,^^35–39^^)^ In Canada, in addition to age, prior fragility fracture status, and femoral neck BMD, major osteoporotic fractures were significantly predicted by BMI, and HFs were predicted by sex as an independent factor.^(^^40^^)^ In contrast, BMI was not found to be an independent predictor in this study. Recently, a few studies have focused on tailoring the FRAX® to the Taiwanese population.^(^^17^^,^^26^^,^^38^^)^ Based on those published studies and this study, the consensus of a practical simplified FRAX® will be reached for Taiwan nationwide adult preventive health services in advance.^(^^41^^)^ Additionally, the decomposition and simplification of FRAX® can serve as a valuable reference not only for the people of Taiwan but also as a module for those countries that are seeking simplified country-specific fracture risk assessments.

This study has several strengths, the primary strength being that it is the first to decompose the FRAX® score and identify key clinical risk factors using real-world population-based survey data. Unlike other studies that generated possible combinations through machine learning,^(^^42^^)^ this study employed actual human data and minimized the derivative data to reach a reasonable and simplified module step-by-step. Most importantly, this study not only recognized the logarithmic correlation between the FRAX® score and clinical risk factors but also used logarithmic transformation to enhance the predictive power of linear regression models. Recently, the FRAXplus® developed by Osteoporosis Research Ltd encouraged the incorporation of more clinical risk factors to predict outcomes in advance using the FRAX®.^(^^19^^)^ In contrast, this study included the integration of real-world data to streamline the original FRAX® and thus provided the processing module as a reference for any country that wishes to develop a simplified bone fracture prediction module to identify high-fracture-risk individuals in a more convenient way.

The main limitation of this study is the lack of long-term follow-up data for real-world fractures collected. This research aimed to deconstruct and streamline the comprehensive FRAX® to enhance its suitability for primary screening. The current module lacks a long-term follow-up analysis of detailed examinations and intervention thresholds. Indeed, we are actively addressing the challenges associated with long-term follow-up studies. Up to now, we have been unable to furnish details on fracture events as the primary outcome of this study. A long-term follow-up cohort is crucial but not always accessible for most countries eager to have their own fracture predictive module at present. Therefore, our findings can lead to the use of a surrogate module, which can be verified by internal and external verification.

Moreover, while smoking and alcohol consumption are recognized risk factors for FRAX®, their prevalence among the Taiwanese elderly population is low (6.6%),^(^^43^^)^ thus reducing their influence in this study. This study aimed to simplify the FRAX® to enhance its suitability for primary screening. Therefore, we used the same threshold (MOF ≥ 20%) for both the full (as a reference) and simplified FRAX® to dichotomize the high- and low-risk subgroups. The simplified FRAX® (without BMD) demonstrated model accuracy exceeding 90%, with a sensitivity of 0.5, a specificity of 0.96, a positive predictive value of 0.43, and a negative predictive value of 0.97. Furthermore, women and older adults had higher odds of incorrect classification. Currently, the intervention threshold of this study still needs to consider factors such as the public health system, cost-effectiveness, expert consensus, and health insurance system. Therefore, the simplified module introduced in this study is a substitute for FRAX® as a starting point. We will keep working on these unmet needs.

In conclusion, in addition to femoral neck BMD, age, sex, and previous fracture history were identified as key predictors of fractures in the decomposed FRAX®. Even in the absence of femoral neck BMD, the simplified FRAX® can be sensibly used as a country-specific calculator for screening 10-year osteoporotic fracture risk in both community and clinical settings.

## Supplementary Material

Supplementary_Table_v8_ziae039

## Data Availability

The data underlying this study are from the National Health Insurance Research Database, Health and Welfare Data Science Center (NHIRD_MOHW).
