# Supplementary material for: Decomposing and simplifying the Fracture Risk Assessment Tool—a module from the Taiwan-specific calculator
Source: JBMR Plus. 2024 Mar 23;8(5):ziae039. doi: 10.1093/jbmrpl/ziae039 (PMC11032218; doi:10.1093/jbmrpl/ziae039)
Supplement: Supplementary_Table_v8_ziae039 [file supplementary_table_v8_ziae039.docx]

Supplemental Table S1.Distribution of participants by different interventional threshold of FRAX^®^ probability (*n* =16384)

|  | IOF ^(28)^ and Taiwanese Osteoporosis Association ^(29)^ | | | Liu et al. ^(26)^ | |
| --- | --- | --- | --- | --- | --- |
|  | High risk | Median risk | Low risk | High risk | Low risk |
| MOF risk | >=20 | 10~19.9 | <10 | >=9.5 | <9.5 |
| with BMD | 925(5.7) | 3474(21.5) | 11728(72.7) | 4783(29.7) | 11344(70.3) |
| without BMD | 1077(6.6) | 4132(25.2) | 11175(68.2) | 5639(34.4) | 10745(65.6) |
| HF risk | >=3 | 1.5~2.9 | <1.5 | >=4.0 | <4.0 |
| with BMD | 4684(29.0) | 3219(20.0) | 8224(51.0) | 3442(21.3) | 12685(78.7) |
| without BMD | 6560(40.0) | 3202(19.5) | 6622(40.4) | 5167(31.5) | 11217(68.5) |

FRAX^®^, Fracture Risk Assessment Tool; BMD, bone mineral density; MOF, major osteoporotic fracture; HF, hip fracture.

Supplemental Table S2. Decomposition of FRAX^®^ with multiple linear regression analysis based on logarithmic 10-year MOF probability strata by sex (*n* =16384)

|  | With BMD | | Without BMD | |
| --- | --- | --- | --- | --- |
|  | Male (*n* =3926) | Female (*n* =12201) | Male (*n* =3983) | Female (*n* =12401) |
|  | β (95% CI) | β (95% CI) | β (95% CI) | β (95% CI) |
| Multiple *R*^2^ value | 0.875 | 0.9407 | 0.9611 | 0.9676 |
| Adjusted *R*^2^ value | 0.8746 | 0.9407 | 0.961 | 0.9675 |
| Femoral neck BMD | −2.55(−2.59-−2.50) | −2.84(−2.87-−2.81) | - | - |
| Age | 0.02(0.02-0.02) | 0.04(0.04-0.04) | 0.05(0.04-0.05) | 0.06(0.06-0.06) |
| Height | −0.01(−0.01-−0.01) | −0.01(−0.01-−0.004) | 0.01(0.01-0.01) | 0.01(0.01-0.01) |
| Weight | 0.01(0.01-0.01) | 0.01(0.01-0.01) | −0.01(−0.01-−0.01) | −0.01(−0.01-−0.01) |
| Parent fractured hip | 0.64(0.61-0.66) | 0.64(0.63-0.65) | 0.63(0.61-0.64) | 0.61(0.60-0.62) |
| Previous fracture | 0.44(0.40-0.47) | 0.45(0.43-0.46) | 0.58(0.55-0.60) | 0.58(0.57-0.60) |
| Glucocorticoids use | 0.44(0.40-0.48) | 0.42(0.40-0.44) | 0.44(0.42-0.46) | 0.46(0.45-0.47) |
| Rheumatoid arthritis | 0.25(0.21-0.29) | 0.28(0.26-0.30) | 0.33(0.30-0.35) | 0.26(0.24-0.27) |
| Secondary osteoporosis | −0.01(−0.04-0.02) | 0.01(−0.003-0.01) | 0.34(0.32-0.35) | 0.32(0.32-0.33) |
| Current smoking | 0.04(0.02-0.06) | 0.03(−0.002-0.06) | 0.07(0.06-0.08) | 0.06(0.03-0.08) |
| Alcohol consumption | 0.28(0.24-0.31) | 0.24(0.20-0.29) | 0.26(0.24-0.28) | 0.26(0.22-0.30) |

FRAX^®^, Fracture Risk Assessment Tool; BMD, bone mineral density; MOF, major osteoporotic fracture.

Supplemental Table S3. Decomposition of FRAX^®^ with multiple linear regression analysis based on logarithmic 10-year HF probability strata by sex (*n* =16384)

|  | With BMD | | Without BMD | |
| --- | --- | --- | --- | --- |
|  | Male (*n* =3926) | Female (*n* =12201) | Male (*n* =3983) | Female (*n* =12401) |
|  | β (95% CI) | β (95% CI) | β (95% CI) | β (95% CI) |
| Multiple *R*^2^ value | 0.9068 | 0.953 | 0.9473 | 0.9608 |
| Adjusted *R*^2^ value | 0.9065 | 0.9529 | 0.9472 | 0.9608 |
| Femoral neck BMD | −6.26(−6.35-−6.17) | −8.29(−8.35-−8.23) |  |  |
| Age | 0.06(0.06-0.06) | 0.06(0.06-0.06) | 0.10(0.10-0.10) | 0.12(0.12-0.12) |
| Height | −0.01(−0.01-−0.004) | −0.001(−0.002-0.00005) | 0.02(0.02-0.02) | 0.02(0.02-0.02) |
| Weight | 0.00(0.00-0.01) | 0.004(0.003-0.004) | −0.02(−0.03-−0.02) | −0.03(−0.03-−0.03) |
| Parent fractured hip | 0.45(0.41-0.50) | 0.32(0.29-0.34) | 0.55(0.52-0.59) | 0.45(0.43-0.47) |
| Previous fracture | 0.42(0.34-0.49) | 0.40(0.38-0.43) | 0.68(0.63-0.74) | 0.75(0.73-0.78) |
| Glucocorticoids use | 0.50(0.43-0.58) | 0.55(0.51-0.58) | 0.64(0.58-0.69) | 0.68(0.65-0.70) |
| Rheumatoid arthritis | 0.29(0.22-0.37) | 0.35(0.32-0.38) | 0.45(0.40-0.51) | 0.41(0.38-0.43) |
| Secondary osteoporosis | −0.02(−0.08-0.04) | 0.01(−0.002-0.03) | 0.49(0.45-0.53) | 0.52(0.51-0.54) |
| Current smoking | 0.41(0.37-0.44) | 0.41(0.35-0.46) | 0.29(0.27-0.32) | 0.31(0.27-0.36) |
| Alcohol consumption | 0.40(0.33-0.46) | 0.35(0.27-0.44) | 0.43(0.39-0.48) | 0.46(0.39-0.53) |

FRAX^®^, Fracture Risk Assessment Tool; BMD, bone mineral density; HF, hip fracture.

Supplemental Table S4. Decomposition of FRAX^®^ with multiple linear regression analysis based on logarithmic 10-year MOF probability strata by age (*n* =16384)

|  | With BMD | | Without BMD | |
| --- | --- | --- | --- | --- |
|  | <65 years old (*n* =7759) | >=65 years old (*n* =8368) | <65 years old (*n* =7908) | >=65 years old (*n* =8476) |
|  | β (95% CI) | β (95% CI) | β (95% CI) | β (95% CI) |
| Multiple *R*^2^ value | 0.953 | 0.9365 | 0.9870 | 0.9509 |
| Adjusted *R*^2^ value | 0.9529 | 0.9364 | 0.9870 | 0.9508 |
| Femoral neck BMD | −2.37(−2.40-−2.35) | −3.08(−3.11-−3.05) | - | - |
| Age | 0.05(0.05-0.06) | 0.01(0.01-0.01) | 0.07(0.07-0.07) | 0.04(0.04-0.04) |
| Sex | 0.18(0.17-0.19) | 0.35(0.34-0.36) | 0.40(0.39-0.40) | 0.66(0.66-0.67) |
| Height | −0.004(−0.004-−0.003) | −0.01(−0.01-−0.01) | 0.01(0.01-0.01) | 0.01(0.01-0.01) |
| Weight | 0.004(0.004-0.004) | 0.01(0.01-0.01) | −0.01(−0.01-−0.01) | −0.01(−0.01-−0.01) |
| Parent fractured hip | 0.65(0.64-0.66) | 0.59(0.57-0.60) | 0.63(0.63-0.64) | 0.57(0.56-0.58) |
| Previous fracture | 0.58(0.56-0.60) | 0.40(0.38-0.41) | 0.74(0.73-0.75) | 0.53(0.51-0.54) |
| Glucocorticoids use | 0.47(0.46-0.49) | 0.39(0.37-0.41) | 0.49(0.49-0.50) | 0.43(0.42-0.45) |
| Rheumatoid arthritis | 0.27(0.26-0.29) | 0.27(0.25-0.29) | 0.25(0.24-0.26) | 0.29(0.28-0.31) |
| Secondary osteoporosis | 0.002(−0.01-0.01) | 0.01(−0.001-0.02) | 0.30(0.30-0.30) | 0.35(0.34-0.36) |
| Current smoking | 0.05(0.04-0.07) | 0.07(0.05-0.09) | 0.08(0.08-0.09) | 0.10(0.08-0.11) |
| Alcohol consumption | 0.23(0.20-0.26) | 0.27(0.23-0.30) | 0.20(0.19-0.22) | 0.28(0.25-0.30) |

FRAX^®^, Fracture Risk Assessment Tool; BMD, bone mineral density; MOF, major osteoporotic fracture.

Supplemental Table S5. Decomposition of FRAX^®^ with multiple linear regression analysis based on logarithmic 10-year HF probability strata by age (*n* =16384)

|  | With BMD | | Without BMD | |
| --- | --- | --- | --- | --- |
|  | <65 years old (*n* =7759) | >=65 years old (*n* =8368) | <65 years old (*n* =7908) | >=65 years old (*n* =8476) |
|  | β (95% CI) | β (95% CI) | β (95% CI) | β (95% CI) |
| Multiple *R*^2^ value | 0.9806 | 0.9318 | 0.9781 | 0.9280 |
| Adjusted *R*^2^ value | 0.9805 | 0.9317 | 0.9781 | 0.9279 |
| Femoral neck BMD | −9.30(−9.34-−9.27) | −6.64(−6.69-−6.59) | - | - |
| Age | 0.08(0.08-0.08) | 0.03(0.03-0.04) | 0.14(0.14-0.14) | 0.08(0.08-0.08) |
| Sex | −0.38(−0.39-−0.36) | −0.11(−0.13-−0.10) | 0.51(0.50-0.52) | 0.58(0.57-0.59) |
| Height | −0.000004(−0.001-0.001) | −0.004(−0.005-−0.003) | 0.03(0.03-0.03) | 0.02(0.02-0.02) |
| Weight | −0.001(−0.001-−0.00004) | 0.004(0.004-0.005) | −0.03(−0.03-−0.03) | −0.03(−0.03-−0.03) |
| Parent fractured hip | 0.03(0.02-0.05) | 0.76(0.74-0.79) | 0.26(0.25-0.27) | 0.73(0.71-0.75) |
| Previous fracture | 0.61(0.58-0.63) | 0.34(0.31-0.36) | 1.12(1.10-1.14) | 0.58(0.56-0.60) |
| Glucocorticoids use | 0.61(0.59-0.63) | 0.50(0.46-0.53) | 0.74(0.73-0.76) | 0.61(0.58-0.63) |
| Rheumatoid arthritis | 0.34(0.32-0.36) | 0.35(0.31-0.39) | 0.41(0.40-0.43) | 0.42(0.39-0.44) |
| Secondary osteoporosis | −0.001(−0.01-0.01) | 0.02(−0.0001-0.04) | 0.51(0.50-0.52) | 0.53(0.51-0.54) |
| Current smoking | 0.48(0.46-0.50) | 0.37(0.33-0.40) | 0.38(0.36-0.40) | 0.27(0.25-0.30) |
| Alcohol consumption | 0.39(0.35-0.43) | 0.36(0.30-0.42) | 0.42(0.39-0.45) | 0.40(0.36-0.44) |

FRAX^®^, Fracture Risk Assessment Tool; BMD, bone mineral density; HF, hip fracture.

Supplemental Table S6. Simplified FRAX^®^ using four premier clinical risk factors with logarithmic 10-year fracture probability (*n* =16384)

|  | Total Participants  (*n* =16384) | | Male  (*n* =3983) | | Female  (*n* =12401) | | <65 years old  (*n* =7908) | | >=65 years old  (*n* =8476) | |
| --- | --- | --- | --- | --- | --- | --- | --- | --- | --- | --- |
|  | MOF risk | HF risk | MOF risk | HF risk | MOF risk | HF risk | MOF risk | HF risk | MOF risk | HF risk |
|  | β (95% CI) | β (95% CI) | β (95% CI) | β (95% CI) | β (95% CI) | β (95% CI) | β (95% CI) | β (95% CI) | β (95% CI) | β (95% CI) |
| Multiple *R*^2^ value | 0.8771 | 0.8655 | 0.8610 | 0.8627 | 0.8886 | 0.8677 | 0.8392 | 0.7482 | 0.7930 | 0.6785 |
| Adjusted *R*^2^ value | 0.8771 | 0.8655 | 0.8609 | 0.8626 | 0.8886 | 0.8677 | 0.8391 | 0.7481 | 0.7929 | 0.6784 |
| Intercept | −1.71  (−1.74-−1.69) | −6.51  (−6.55-−6.46) | −1.41  (−1.45-−1.37) | −6.60  (−6.69-−6.51) | −1.97  (−2.00-−1.95) | −6.64  (−6.70-−6.59) | −2.47  (−2.51-−2.42) | −7.46  (−7.55-−7.36) | −0.46  (−0.52-−0.40) | −4.19  (−4.29-−4.08) |
| Age | 0.06  (0.06-0.06) | 0.11  (0.11-0.11) | 0.04  (0.04-0.05) | 0.10  (0.10-0.11) | 0.06  (0.06-0.06) | 0.11  (0.11-0.11) | 0.07  (0.07-0.07) | 0.13  (0.13-0.13) | 0.04  (0.04-0.04) | 0.08  (0.08-0.08) |
| Sex | −0.60  (−0.61-−0.59) | −0.59  (−0.61-−0.57) | - | - | - | - | −0.44  (−0.46-−0.43) | −0.58  (−0.61-−0.56) | −0.69  (−0.70-−0.68) | −0.60  (−0.62-−0.58) |
| Parent fractured hip | 0.69  (0.67-0.70) | 0.59  (0.56-0.62) | 0.70  (0.68-0.73) | 0.66  (0.61-0.72) | 0.68  (0.66-0.70) | 0.57  (0.54-0.61) | 0.70  (0.69-0.72) | 0.41  (0.37-0.44) | 0.63  (0.61-0.66) | 0.84  (0.80-0.88) |
| Previous fracture | 0.67  (0.65-0.70) | 0.87  (0.83-0.91) | 0.66  (0.62-0.70) | 0.82  (0.74-0.91) | 0.66  (0.64-0.69) | 0.87  (0.82-0.91) | 0.82  (0.79-0.85) | 1.24  (1.16-1.31) | 0.61  (0.58-0.63) | 0.70  (0.65-0.74) |

FRAX^®^, Fracture Risk Assessment Tool; MOF, major osteoporotic fracture; HF, hip fracture.

Supplemental Table S7. Simplified FRAX^®^ using three premier clinical risk factors and femoral neck BMD with logarithmic 10-year fracture probability (*n* =16127)

|  | Total Participants  (*n* =16127) | | Male  (*n* =3926) | | Female  (*n* =12201) | | <65 years old  (*n* =7759) | | >=65 years old  (*n* =8368) | |
| --- | --- | --- | --- | --- | --- | --- | --- | --- | --- | --- |
|  | MOF risk | HF risk | MOF risk | HF risk | MOF risk | HF risk | MOF risk | HF risk | MOF risk | HF risk |
|  | β (95% CI) | β (95% CI) | β (95% CI) | β (95% CI) | β (95% CI) | β (95% CI) | β (95% CI) | β (95% CI) | β (95% CI) | β (95% CI) |
| Multiple *R*^2^ value | 0.8263 | 0.9132 | 0.7087 | 0.8644 | 0.8539 | 0.9328 | 0.7857 | 0.9423 | 0.8487 | 0.8809 |
| Adjusted *R*^2^ value | 0.8262 | 0.9131 | 0.7085 | 0.8643 | 0.8538 | 0.9328 | 0.7856 | 0.9423 | 0.8486 | 0.8808 |
| Intercept | 1.68  (1.63-1.73) | 1.48  (1.41-1.54) | 2.15  (2.06-2.23) | 1.03  (0.89-1.16) | 1.40  (1.35-1.46) | 1.92  (1.85-1.99) | 0.19  (0.11-0.26) | 1.89  (1.81-1.97) | 3.62  (3.55-3.69) | 3.00  (2.89-3.12) |
| Age | 0.03  (0.03-0.03) | 0.06  (0.06-0.06) | 0.02  (0.02-0.02) | 0.06  (0.05-0.06) | 0.04  (0.04-0.04) | 0.06  (0.06-0.06) | 0.05  (0.05-0.06) | 0.07  (0.07-0.08) | 0.01  (0.01-0.01) | 0.03  (0.03-0.03) |
| Sex | −0.32  (−0.33-−0.30) | 0.28  (0.26-0.30) | - | - | - | - | −0.17  (−0.19-−0.16) | 0.46  (0.44-0.47) | −0.37  (−0.38-−0.36) | 0.13  (0.11−0.15) |
| Previous fracture | 0.57  (0.54-0.59) | 0.52  (0.49-0.56) | 0.58  (0.52-0.63) | 0.58  (0.49-0.67) | 0.55  (0.52-0.57) | 0.50  (0.47-0.53) | 0.70  (0.66-0.74) | 0.70  (0.66-0.75) | 0.50  (0.47-0.52) | 0.47  (0.43-0.51) |
| Femoral neck BMD | −2.74  (−2.78-−2.70) | −7.63  (−7.69-−7.57) | −2.43  (−2.49-−2.36) | −6.24  (−6.35-−6.14) | −2.77  (−2.82-−2.73) | −8.26  (−8.33-−8.20) | −2.40  (−2.46-−2.35) | −9.43  (−9.49-−9.36) | −2.98  (−3.03-−2.94) | −6.61  (−6.68-−6.55) |

FRAX^®^, Fracture Risk Assessment Tool; BMD, bone mineral density; MOF, major osteoporotic fracture; HF, hip fracture.

Supplemental Table S8. Simplified FRAX^®^ using four premier clinical risk factors and femoral neck BMD with logarithmic 10-year fracture probability (*n* =16127)

|  | Total Participants  (*n* =16127) | | Male  (*n* =3926) | | Female  (*n* =12201) | | <65 years old  (*n* =7759) | | >=65 years old  (*n* =8368) | |
| --- | --- | --- | --- | --- | --- | --- | --- | --- | --- | --- |
|  | MOF risk | HF risk | MOF risk | HF risk | MOF risk | HF risk | MOF risk | HF risk | MOF risk | HF risk |
|  | β (95% CI) | β (95% CI) | β (95% CI) | β (95% CI) | β (95% CI) | β (95% CI) | β (95% CI) | β (95% CI) | β (95% CI) | β (95% CI) |
| Multiple *R*^2^ value | 0.8790 | 0.8710 | 0.8088 | 0.8756 | 0.9028 | 0.9364 | 0.8821 | 0.9427 | 0.896 | 0.9084 |
| Adjusted *R*^2^ value | 0.8789 | 0.8710 | 0.8086 | 0.8755 | 0.9028 | 0.9364 | 0.8821 | 0.9426 | 0.8959 | 0.9083 |
| Intercept | 1.56  (1.52-1.59) | 1.65  (1.61-1.68) | 2.01  (1.94-2.09) | 0.92  (0.79-1.05) | 1.28  (1.24-1.32) | 1.85  (1.78-1.92) | 0.14  (0.08-0.20) | 1.88  (1.80-1.97) | 3.49  (3.43-3.55) | 2.83  (2.73-2.93) |
| Age | 0.03  (0.03-0.03) | 0.03  (0.03-0.03) | 0.02  (0.02-0.02) | 0.06  (0.06-0.06) | 0.04  (0.04-0.04) | 0.06  (0.06-0.06) | 0.05  (0.05-0.06) | 0.07  (0.07-0.08) | 0.01  (0.01-0.01) | 0.03  (0.03-0.04) |
| Sex | −0.32  (−0.33-−0.31) | −0.32  (−0.33-−0.31) | - | - | - | - | −0.18  (−0.20-−0.17) | 0.45  (0.44-0.47) | −0.38  (−0.39-−0.37) | 0.13  (0.11-0.14) |
| Parent fractured hip | 0.67  (0.65-0.68) | 0.66  (0.65-0.68) | 0.69  (0.66-0.72) | 0.52  (0.46-0.57) | 0.65  (0.64-0.67) | 0.34  (0.32-0.37) | 0.67  (0.66-0.69) | 0.08  (0.06-0.10) | 0.61  (0.59-0.63) | 0.80  (0.76-0.83) |
| Previous fracture | 0.51  (0.49-0.54) | 0.51  (0.49-0.53) | 0.51  (0.47-0.56) | 0.53  (0.45-0.61) | 0.50  (0.48-0.52) | 0.47  (0.44-0.51) | 0.65  (0.62-0.68) | 0.70  (0.65-0.74) | 0.45  (0.43-0.47) | 0.40  (0.37-0.44) |
| Femoral neck BMD | −2.72  (−2.75-−2.68) | −2.81  (−2.84-−2.78) | −2.42  (−2.47-−2.37) | −6.24  (−6.35-−6.14) | −2.75  (−2.78-−2.71) | −8.25  (−8.31-−8.19) | −2.37  (−2.41-−2.33) | −9.42  (−9.49-−9.36) | −2.98  (−3.01-−2.94) | −6.60  (−6.66-−6.55) |

FRAX^®^, Fracture Risk Assessment Tool; BMD, bone mineral density; MOF, major osteoporotic fracture; HF, hip fracture.

Supplemental Table S9. Correlation and Collinearity Diagnostics of FRAX^®^ risk factor (*n* =16384)

|  | MOF risk | | HF risk | | VIF | |
| --- | --- | --- | --- | --- | --- | --- |
|  | with BMD | without BMD | with BMD | without BMD | with BMD | without BMD |
| Femoral neck BMD | −0.782 |  | −0.847 |  | 1.4758 |  |
| Age | 0.645 | 0.811 | 0.713 | 0.905 | 1.1293 | 1.0184 |
| Height | −0.432 | −0.341 | −0.320 | −0.235 | 1.4556 | 1.3828 |
| Weight | −0.257 | −0.256 | −0.286 | −0.269 | 1.5206 | 1.3617 |

FRAX^®^, Fracture Risk Assessment Tool; BMD, bone mineral density; MOF, major osteoporotic fracture; HF, hip fracture; VIF, variance inflation factor.

Supplemental Table S10. The results of external validation in the two cohorts

1. The demographic characteristics in the two cohorts: aged between 40 and 90 years

|  | This study cohort (*n*=16384) | Compatible cohort (*n*=1970) |
| --- | --- | --- |
| Age, years | 64.85±10.58 | 65.30±11.99 |
| Sex |  |  |
| Men | 3983(24.3) | 928(47.1) |
| Women | 12401(75.7) | 1042(52.9) |
| Previous fracture (%) | 518(3.2) | 200(10.2) |
| FRAX^®^ without BMD, % |  |  |
| MOF risk | 9.07±6.63 | 9.96±7.88 |
| HF risk | 3.63±4.54 | 4.30±5.04 |

1. Simplified FRAX^®^ using three premier clinical risk factors with logarithmic 10-year fracture probability in the two cohorts

|  | This study cohort (*n*=16384) | | Compatible cohort (*n*=1970) | |
| --- | --- | --- | --- | --- |
|  | MOF risk | HF risk | MOF risk | HF risk |
|  | β (95% CI) | β (95% CI) | β (95% CI) | β (95% CI) |
| Adjusted *R*^2^ value | 0.82 | 0.85 | 0.66 | 0.62 |
| Intercept | −1.61(−1.63-−1.58) | −6.41(−6.46-−6.37) | −1.64(−1.77-−1.51) | −5.16(−5.40-−4.92) |
| Age | 0.06(0.06-0.06) | 0.11(0.11-0.11) | 0.05(0.04-0.05) | 0.08(0.08-0.09) |
| Sex | −0.60(−0.61-−0.58) | −0.59(−0.61-−0.57) | 0.45(0.41-0.48) | 0.23(0.16-0.30) |
| Previous fracture | 0.73(0.70-0.75) | 0.91(0.87-0.95) | 0.58(0.52-0.64) | 0.71(0.59-0.82) |
